# Autoimmunity in Wiskott–Aldrich Syndrome: An Unsolved Enigma

**DOI:** 10.3389/fimmu.2012.00209

**Published:** 2012-07-18

**Authors:** Marco Catucci, Maria Carmina Castiello, Francesca Pala, Marita Bosticardo, Anna Villa

**Affiliations:** ^1^San Raffaele Telethon Institute for Gene Therapy (HSR-TIGET)Milan, Italy; ^2^Vita-Salute San Raffaele UniversityMilan, Italy; ^3^Milan Unit, Istituto di Ricerca Genetica e Biomedica, Consiglio Nazionale delle RicercheMilan, Italy

**Keywords:** Wiskott–Aldrich syndrome, autoimmunity, primary immunodeficiency, T lymphocytes, B lymphocytes

## Abstract

Wiskott–Aldrich Syndrome (WAS) is a severe X-linked Primary Immunodeficiency that affects 1–10 out of 1 million male individuals. WAS is caused by mutations in the WAS Protein (WASP) expressing gene that leads to the absent or reduced expression of the protein. WASP is a cytoplasmic protein that regulates the formation of actin filaments in hematopoietic cells. WASP deficiency causes many immune cell defects both in humans and in the WAS murine model, the *Was*^−/−^ mouse. Both cellular and humoral immune defects in WAS patients contribute to the onset of severe clinical manifestations, in particular microthrombocytopenia, eczema, recurrent infections, and a high susceptibility to develop autoimmunity and malignancies. Autoimmune diseases affect from 22 to 72% of WAS patients and the most common manifestation is autoimmune hemolytic anemia, followed by vasculitis, arthritis, neutropenia, inflammatory bowel disease, and IgA nephropathy. Many groups have widely explored immune cell functionality in WAS partially explaining how cellular defects may lead to pathology. However, the mechanisms underlying the occurrence of autoimmune manifestations have not been clearly described yet. In the present review, we report the most recent progresses in the study of immune cell function in WAS that have started to unveil the mechanisms contributing to autoimmune complications in WAS patients.

## Wiskott–Aldrich Syndrome: Cellular Defects and Clinical Manifestations

**Figure 1:**
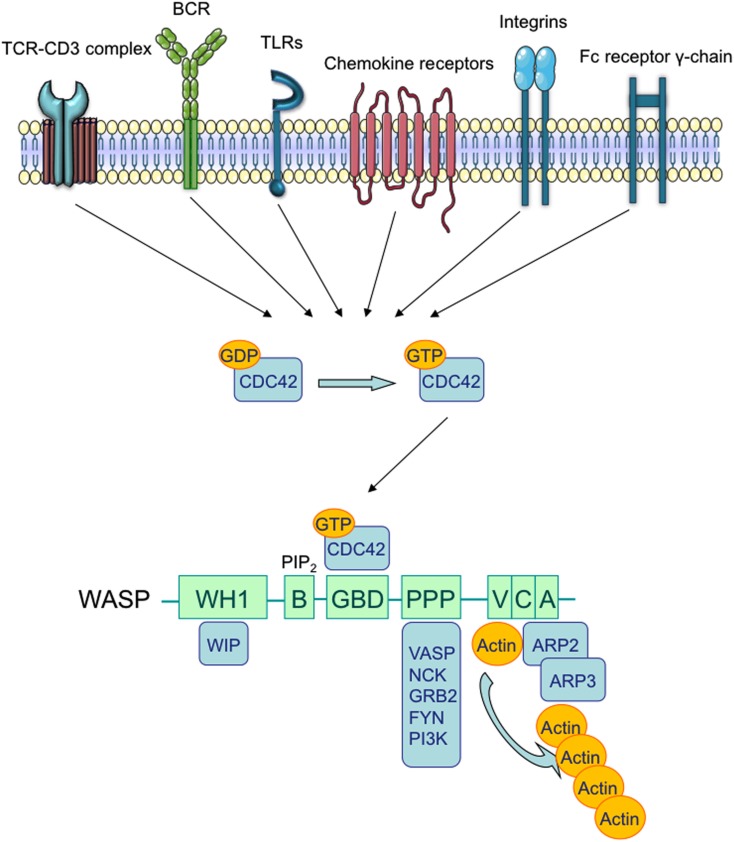
**Wiskott–Aldrich syndrome structure and interacting proteins**. TCR, BCR, chemokine receptors, TLRs, integrins, and the Fc receptor γ-chain can promote the release of GDP from Rho family GTPases, allowing GTP to bind. In immune cells, the major Rho GTPase is the Cell Division Cycle 42 (CDC42). The WASP-Homology 1 (WH1) domain mediates the binding to WASP-Interacting Protein (WIP; Ramesh et al., 1997). The Phosphatidylinositol-4,5-bisphosphate (PIP_2_) links to the Basic (B) domain and stabilizes WASP active form. The binding of the GTPase-Binding Domain (GBD) with CDC42 induces WASP activation (Kolluri et al., 1996; Symons et al., 1996; Miki et al., 1998). The proline-rich region (PPP) provides binding sites for the Vasodilator-Stimulated Phosphoprotein (VASP), and also for SRC family tyrosine kinases and SRC Homology 3 (SH3) domain-containing proteins such as the adaptor proteins GRB2, FYN, PI3K, and NCK. The Verprolin-homology (V) domain binds to actin monomers, and the Cofilin-homology (C) and Acidic (A) domains bind to the Actin-Related Protein (ARP)2/3 complex. The V/C/A region functions as the platform to initiate actin polymerization (Park et al., 2010).

Wiskott–Aldrich Syndrome (WAS) is a rare X-linked Primary Immunodeficiency (PID) that affects 1–10 out of a million male individuals (Ochs and Thrasher, 2006), whose life expectancy is about 15 years in severe cases (Imai et al., 2004). Affected patients demonstrate both cellular and humoral immunodeficiency, high susceptibility to infections, eczema, microthrombocytopenia, and increased risk of autoimmune disorders and lymphomas (Bosticardo et al., 2009). WAS is caused by defective expression of WAS Protein (WASP), a key regulator of cytoskeletal organization in hematopoietic cells (Figure 1). The *WAS* gene is located on the X chromosome and encodes a 502 amino acid protein (Derry et al., 1994), which is constitutively expressed in the cytoplasm of hematopoietic cells (Kim et al., 2000). WASP is present in an auto-inhibited conformation and its activation is mainly induced by the binding with GTPase Cell division Cycle 42 (CDC42; Abdul-Manan et al., 1999). Other factors, such as the Non-Catalytic region of tyrosine Kinase adaptor protein (NCK; Tomasevic et al., 2007), and the phosphorylation of WASP tyrosine residue 291 (Y291) can activate WASP independently of CDC42 (Cory et al., 2002; Badour et al., 2004). The binding of Phosphatidylinositol-4,5-bisphosphate (PIP_2_) is also an important regulator of WASP activation by inducing a stable acting form (Imai et al., 1999). WASP, in the active form, binds the Actin-Related Protein (ARP)2/3 complex, which gives rise to nucleation of actin filaments at the side of pre-existing filaments, thus creating a branching network of actin at the plasma membrane (Symons et al., 1996; Machesky and Insall, 1998; Miki et al., 1998; Machesky and Gould, 1999; Blanchoin et al., 2000; Pantaloni et al., 2000). The activity of the ARP2/3 complex was shown to contribute to a variety of cellular functions, including change of cell shape, motility, endocytosis, and phagocytosis (Welch and Mullins, 2002).

The severity of disease, measured on the basis of the classification proposed by Zhu et al. (1997) and subsequently modified (Ochs and Thrasher, 2006; Ochs et al., 2009), is schematically reported in Table 1.

**Table 1:** **WAS scoring system according to Zhu et al. (1997), with subsequent refinements (Ochs and Thrasher, 2006; Ochs et al., 2009)**.

Clinical scores	iXLT	XLT		WAS		
	<1	1	2	3	4	5
Thrombocytopenia	−/+	+	+	+	+	+
Small platelets	+	+	+	+	+	+
Eczema	−	−	(+)	+	++	(+)/+/++
Immunodeficiency	−	−/(+)	(+)	+	+	(+)/+
Infections	−	−	(+)	+	+/++	(+)/+/++
Autoimmunity or malignancy	−	−	−	−	−	+

*Scoring system: −, absent; (+), mild; +, present; ++, present and severe. iXLT, intermittent X-linked thrombocytopenia; WAS, Wiskott–Aldrich syndrome; XLT, X-linked thrombocytopenia*.

A score from one to two identifies patients affected from a milder form of the disease, named X-Linked Thrombocytopenia (XLT; Villa et al., 1995), and characterized by reduced expression of full-length mutated protein and microthrombocytopenia. Localized eczema and occasional respiratory infections, in addition to microthrombocytopenia, identify score 2 of the disease. Patients who develop microthrombocytopenia, associated with persistent but therapy-responsive eczema or infections receive a score of 3, whereas a score of 4 is given if eczema or infections do not respond to treatments. Finally, score 5 is assigned to patients developing autoimmunity or tumors.

Wiskott–Aldrich Syndrome gene mutations are scattered throughout the entire length of the *WAS* gene, although some hot spots have been identified (Ochs and Thrasher, 2006). Mutations that abolish WASP expression are mainly associated with a severe clinical phenotype (full blown WAS) and a life expectancy below 20 years of age (Jin et al., 2004). On the contrary, missense mutations, which result in residual expression of a full-length point-mutated WASP, are often associated with XLT (Villa et al., 1995; Notarangelo et al., 2002; Albert et al., 2010), corresponding to a disease score of 0.5–2 and a longer life expectancy (Imai et al., 2004). All patients harboring mutations in the *WAS* gene are micro-thrombocytopenic, although intermittent X-Linked Thrombocytopenia (iXLT) is observed in some patients with substantial protein expression (Notarangelo et al., 2002). Importantly, up to 11% of patients can present somatic mosaicism due to spontaneous *in vivo* reversion of the original mutation or second-site compensatory mutations that restore production of the *WAS* gene product (Stewart et al., 2007). The revertant mutation can occur at various stages of hematopoietic differentiation thus conferring high selective advantage to revertant cells over mutated cell populations not expressing WASP. Although many reports describe the occurrence of this phenomenon, it is still not clear whether the presence of somatic mosaicism might correlate with a better clinical course of the disease (Davis and Candotti, 2009; Trifari et al., 2010).

**Figure 2:**
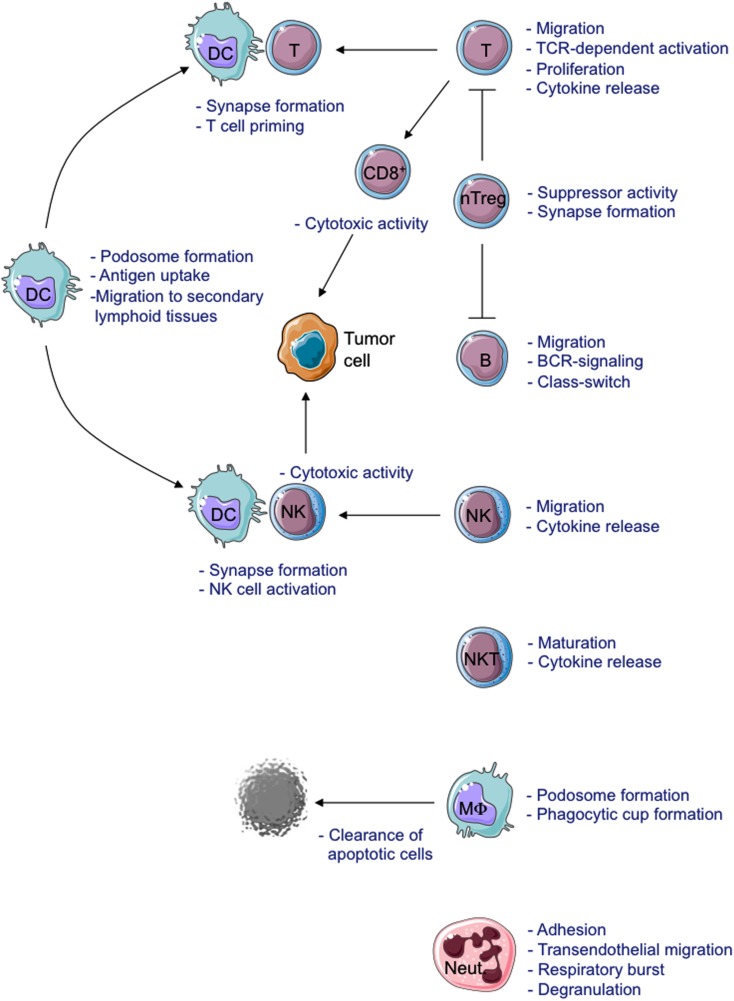
**Schematic view of cellular defects described in WASP-deficient cells**. MΦ, Macrophage; Neut., Neutrophil.

Absence or residual WASP expression causes functional defects in all immune cells (Figure 2).

**Figure 3:**
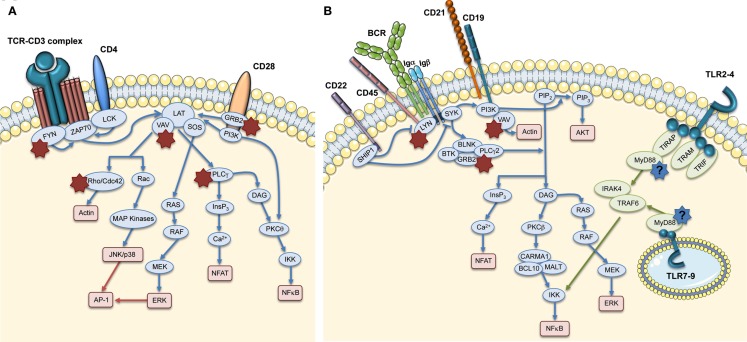
**Role of WASP in TCR and BCR signaling pathways**. The main signaling molecules (blue and green ovals) acting downstream of TCR **(A)**, BCR, and TLRs **(B)** are depicted. The red boxes indicate the main pathways induced downstream of these receptors. The role of WASP in these pathways is indicated by stars close to interacting molecules: red stars show the interactions that are demonstrated, whereas the blue stars with question marks show hypothetical involvement of WASP in TLR signaling pathways.

The formation of the Immunological Synapse (IS) in T cells and T Cell Receptor (TCR)-dependent activation (Dupre et al., 2002; Trifari et al., 2006; Nikolov et al., 2010), the cytotoxic activity of CD8^+^ T cells and Natural Killer (NK) cells (Orange et al., 2002; de Meester et al., 2010) and the suppressor activity of Naturally occurring Regulatory T (nTreg) cells (Adriani et al., 2007, 2011; Humblet-Baron et al., 2007; Maillard et al., 2007; Marangoni et al., 2007) are all impaired in WASP-deficient cells. Motility, adhesion and migration of B cells are also defective (Westerberg et al., 2005; Meyer-Bahlburg et al., 2008). Additionally, the lack of WASP affects podosome formation (Burns et al., 2001; Calle et al., 2004), motility (Binks et al., 1998; de Noronha et al., 2005) and T cell priming in Dendritic Cells (DCs; Pulecio et al., 2008; Bouma et al., 2011), as well as podosome and phagocytic cup formation in macrophages (Linder et al., 1999; Tsuboi and Meerloo, 2007). Invariant Natural Killer T (iNKT) cell functionality (Astrakhan et al., 2009; Locci et al., 2009), adhesion, and migration of neutrophils (Zhang et al., 2006) are also altered in the absence of WASP. Moreover, WASP is also involved in signal transduction (Figure 3). In particular, TCR-dependent nuclear recruitment of Nuclear Factor of Activated T cells (NFAT)-1 in CD4^+^ T cells and both NFAT-1 and NFAT-2 in CD8^+^ T cells are reduced in WAS patients and correlate with defective Th1 cytokine production (Cianferoni et al., 2005; Trifari et al., 2006). Additionally, WASP is involved in B Cell Receptor (BCR) signaling by binding to the Src homology three domains of several tyrosine kinases, such as the Bruton’s Tyrosine Kinase (BTK; Cory et al., 1996; Sharma et al., 2009).

The most common finding in WAS patients is microthrombocytopenia which causes frequent hemorrhages in more than 80% of patients (Ochs, 2002; Imai et al., 2004) and severe bleeding episodes that lead to death in 4–10% of patients (Sullivan et al., 1994; Imai et al., 2004). The mechanism underlying thrombocytopenia is not completely understood. One possible explanation could be an abnormal platelet clearance induced by an increased exposure of phosphatidylserine on the outer plasma membrane of WASP-deficient platelets (Shcherbina et al., 2009). Another mechanism of platelet reduction that needs to be investigated more in detail, is the elimination mediated by autoimmune reaction. In fact, the presence of platelet-associated antibodies in *Was*^−/−^ mice (Marathe et al., 2009) and in some patients has been reported (Corash et al., 1985; Semple et al., 1997). The second most common manifestation in WAS patients is the eczema. It is observed in 80% of patients and its severity inversely correlates with the expression of WASP. Indeed, it has been shown that WAS patients with residual WASP expression develop moderate or transient form of the disease, whereas most of WASP-negative patients develop severe, treatment-resistant eczema (Imai et al., 2004). High IgE levels (more than 1000 IU/mL) were observed in 62% of WASP-negative patients and in 25% of WASP-positive. Although higher IgE levels may represent a possible cause of eczema, the correlation between increased IgE levels and eczema has not yet been demonstrated. WAS patients are highly susceptible to infections by bacteria, viruses, and fungi (Imai et al., 2004). Of note, WASP-negative patients are more frequently affected by bacterial infections (otitis media, skin abscess, pneumonia, enterocolitis, meningitis, sepsis, urinary tract infection, and others), viral infections (*Herpes simplex* and *Cytomegalovirus*) and fungal infections (*Candida* spp., *Aspergillus* spp., and *Pneumocystis carinii*) as compared to WASP-positive patients (Imai et al., 2004). Patients with clinically most severe WAS develop malignancies and/or autoimmune manifestations. Malignancies can affect adolescent and young adult WAS patients more than infants (Sullivan et al., 1994; Imai et al., 2004). Epstein–Barr virus (EBV)-positive B cell lymphoma is most frequently reported, but also myelodysplasia can be observed in some patients (Imai et al., 2004). Autoimmune complications are frequently observed in WAS, affecting 22–72% of patients (Dupuis-Girod et al., 2003; Imai et al., 2004). WAS patients with autoimmune diseases constitute a high-risk group with poor prognosis. Moreover, autoimmunity is associated with a higher risk of a later development of tumors and an increased risk of mortality (Sullivan et al., 1994). A better understanding of the mechanisms underlying autoimmunity in WAS would be crucial for the development of more effective therapies for the management of these manifestations in WAS and could also provide new insights in the pathogenesis of autoimmunity in PIDs.

## Autoimmune Manifestations in WAS Patients and Current Treatments

The most common autoimmune manifestation in WAS is hemolytic anemia (36%), followed by vasculitis (including cerebral vasculitis; 29%), arthritis (29%), neutropenia (25%), inflammatory bowel disease (9%), and IgA nephropathy (3%). Henoch–Schönlein-like purpura, dermatomyositis, recurrent angioedema, and uveitis have also been reported in some patients (Dupuis-Girod et al., 2003; Imai et al., 2004). Moreover, multiple autoimmune manifestations can be observed. In most cases, and in all cases of hemolytic anemia, the onset of autoimmune complications occurs early in life (0–5 years; Dupuis-Girod et al., 2003). Interestingly, it has been recently found that *Was*^−/−^ mice develop proliferative glomerulonephritis with increased IgA in the serum and IgA production by splenic B cells (Nikolov et al., 2010; Shimizu et al., 2012). Moreover old *Was*^−/−^ mice showed aberrant glycosylation of IgA (Shimizu et al., 2012), feature that has been associated to the development of nephropathy-like glomerular lesions with IgA deposition (Nishie et al., 2007). Although these studies have been performed on WAS mouse model, they clearly suggest a possible mechanism for the pathogenesis of glomerulonephritis in WAS patients.

Clinical management of WAS patients is a significant challenge since, with the exception of Bone Marrow Transplantation (BMT), most available therapies are not curative. Intravenous immunoglobulins (IVIG) and antibiotic prophylaxis are often used to reduce the risk of infections in WAS patients, but it is not clear whether these treatments effectively reduce the incidence of life-threatening infections (Conley et al., 2003). Splenectomy significantly increases and often normalizes the platelet counts (Corash et al., 1985; Mullen et al., 1993). However, it does not fully overcome the risk of bleeding and further predisposes to sepsis, obliging the patients to life-long antibiotic prophylaxis (Mullen et al., 1993). Relapse of thrombocytopenia has been described in a fraction of splenectomized WAS patients (Corash et al., 1985; Dupuis-Girod et al., 2003). Of note, in some cases, thrombocytopenia was found to be autoantibody-mediated and also associated with hemolytic anemia or cerebral vasculitis (Dupuis-Girod et al., 2003). Therefore, splenectomy is indicated only in severe cases, for which there is no prospect for other curative interventions. Treatment with human recombinant Interleukin 2 (hrIL-2) appeared to be effective in reducing herpes virus infections and improving dermatitis in a WAS patient (Azuma et al., 1993). Administration of hrIL-2 ameliorated proliferation of cultured T cells from one patient (Azuma et al., 2000) and restored cytotoxicity and actin accumulation at the IS in NK cells from another treated patient (Orange et al., 2011). Since WAS T cells are less efficient in producing IL-2, NK cells do not receive sufficient IL-2, thus resulting in reduced NK activation and failure to respond effectively to infections. A clinical trial with hrIL-2 is currently ongoing in WAS (ClinicalTrials.gov identifier NCT00774358). The treatment of choice for autoimmune manifestations in WAS patients consists of steroids, alone, or in association with cyclosporine (Dupuis-Girod et al., 2003). Steroids are the first-line treatment for all patients with hemolytic anemia and efficiently induce remission in 10% of cases, are partially effective in 60% of cases, while are ineffective in 30% of cases. Cyclophosphamide and azathioprine are also used in some cases and are effective in a small percentage of cases. Patients with severe autoimmune thrombocytopenia after splenectomy are usually treated with IVIG, high-dose steroids, azathioprine, and cyclophosphamide. Other autoimmune or inflammatory complications are generally treated with steroids, in association with cyclosporine, and are effective in the majority of skin vasculitis, arthritis, bowel inflammatory disease and renal disease cases (Dupuis-Girod et al., 2003). Anti-CD20 monoclonal antibody therapy has been also performed for the treatment of autoimmune hemolytic anemia in some patients. This treatment results effective in correcting the anemia, but it may need repeated courses due to relapse of the disease (Ship et al., 2002; Kim et al., 2007).

Currently, the only resolutive therapeutic option for WAS patients is BMT. When a Related HLA-Identical Donor (RID) is available, BMT leads to 73–100% survival (Mullen et al., 1993; Ozsahin et al., 1996, 2008; Filipovich et al., 2001; Antoine et al., 2003; Kobayashi et al., 2006; Pai et al., 2006; Moratto et al., 2011). On the other hand, transplantation using the bone marrow of a Mismatched Related Donor (MMRD) results in a poor survival ranging from 29 to 52% (Mullen et al., 1993; Filipovich et al., 2001; Kobayashi et al., 2006; Ozsahin et al., 2008). In addition, this type of transplant is associated with an elevated risk of developing life-threatening EBV^+^ lymphoproliferative syndrome, infections, autoimmunity, and graft-versus-host disease (GVHD; Filipovich et al., 2001), therefore it is not recommended except in case of emergency. When a suitable related donor is missing, transplantation using the bone marrow or cord blood from a Matched Unrelated Donor (MUD) is a valid therapeutic option, leading to 71–81% survival (Filipovich et al., 2001; Kobayashi et al., 2006; Pai et al., 2006). Two recent retrospective studies have analyzed long-term outcome and donor cell engraftment in WAS patients who have been treated by Hematopoietic Stem Cell Transplantation (HSCT; Ozsahin et al., 2008; Moratto et al., 2011). They observed that 20% of patients developed autoimmune manifestations after HSCT independently of chronic GVHD (Ozsahin et al., 2008) and some patients had more than one manifestation. Autoimmune manifestations appeared at a median of 1.5 years after HSCT (range: 4 months to 10 years). The median duration of autoimmunity was 4 years (range: 1–20 years). Autoimmune manifestations were more frequent in recipients of MUD (28%) and MMRD (26%) than RID HSCT (11%). Ozsahin and colleagues investigated whether patients developing autoimmunity after HSCT had autoimmune manifestations also before treatment. Overall, 17 patients had autoimmune manifestations before transplantation that persisted thereafter in seven of them. Conversely, autoimmunity occurred *de novo* in 11–23% of transplanted patients. A very interesting observation in both retrospective studies was the strong correlation between autoimmunity occurrence and the chimerism pattern. Overall, incomplete reconstitution of lymphocyte counts and incidence of autoimmunity were higher in patients with a lower degree of chimerism in both lymphoid and myeloid compartments as compared to patients with full chimerism (Ozsahin et al., 2008; Moratto et al., 2011).

A very promising alternative to HSCT, when a matched donor is missing, is the infusion of gene corrected autologous Hematopoietic Stem Cells (HSCs). Two different Gene Therapy (GT) clinical trials have been approved: a Retroviral Vector (RV)-mediated gene transfer (Boztug et al., 2010) and a Lentiviral Vector (LV)-mediated GT approach, developed by our and other groups (Dupre et al., 2006; Galy et al., 2008). In the RV-mediated clinical trial, sustained expression of WASP in HSCs, lymphoid and myeloid cells, and platelets was shown in two treated patients 3 years after GT (Boztug et al., 2010). T and B cells, NK cells, and monocytes were also functionally corrected resulting in improved clinical conditions. Signs and symptoms of autoimmunity disappeared in both patients within the first year after GT. In one of the two reported patients, severe autoimmune hemolytic anemia, autoimmune thrombocytopenia, and autoimmune neutropenia disappeared; whereas severe eczema resolved in the second patient. However, in this trial, leukemia occurred in one out of ten GT patients, probably due to insertional mutagenesis caused by RV integration (Press Release, Hannover Medical School, http://www.asgct.org/UserFiles/file/Genetherapy_WAS_final_english.pdf). This adverse event gives rise to some concerns on the safety of RV-mediated GT for WAS. A multicenter clinical trial using a third generation LV carrying *WAS* gene driven by the endogenous promoter is on going in Milan, Paris, and London. Preclinical data in the murine model indicate that the LV-mediated GT approach is effective in restoring immune cell functionality (Blundell et al., 2008; Marangoni et al., 2009; Bosticardo et al., 2011; Catucci et al., 2011). GT treated *Was*^−/−^ mice did not show any adverse events or tumors even in long-term follow up studies (Marangoni et al., 2009). Finally, we and others demonstrated the efficacy of LV-mediated GT in CD34^+^ cells obtained from WAS patients (Charrier et al., 2007; Scaramuzza et al., 2012). Nevertheless, data from the clinical study are needed to provide definitive evidence of the efficacy and safety of this novel therapeutic approach.

## Regulation of T Cell Tolerance in WAS

T cells are significantly reduced in peripheral blood of WAS patients and show a defective proliferation in response to TCR stimulation by CD3-specific antibody, although this defect is present only at low doses of agonistic antibody (Molina et al., 1992, 1993). TCR-dependent activation in WASP-deficient T cells results in a reduced IL-2 production (Molina et al., 1993), that is associated with delayed NFAT-1 nuclear translocation and defective T-bet induction (Cianferoni et al., 2005; Trifari et al., 2006; Taylor et al., 2010). T cell activation is regulated by the formation of the IS, a polarized cluster of TCR, costimulatory molecules, signaling molecules, and integrins at the T cell:antigen presenting cell (APC) interface. To promote their lateral movement on the plasma membrane, the molecules being recruited to the IS are associated with specific cholesterol-enriched membrane microdomains, called lipid rafts. In the absence of WASP, IS can be formed only after strong TCR stimulation (Cannon and Burkhardt, 2004). In particular, WASP-deficient T cells fail to upregulate GM1 on the cell surface, cluster GM1 in the lipid rafts during IS formation (Dupre et al., 2002) and maintain IS stability after migration (Sims et al., 2007).

It is commonly assumed that autoimmunity is a consequence of the breakdown of central or peripheral tolerance to self-antigens. nTreg cells are fundamental to maintain tolerance to self-antigens and suppress excessive immune responses. nTreg cell development and function depend on TCR signaling, together with CD28 recruitment, FOXP3 expression, and presence of IL-2 (Sakaguchi et al., 2008). Several groups, including ours, have described the defects of nTreg cells in WAS patients and *Was*^−/−^ mice in localizing and suppressing T effector cell response (Adriani et al., 2007; Maillard et al., 2007; Marangoni et al., 2007), although their number in blood of WAS patients is comparable with healthy donors (Marangoni et al., 2007). It is not clear whether a defective thymic development of *Was*^−/−^ nTreg cells could account for their impaired *in vivo* suppressive function, since one group has shown reduced nTreg cell percentage in the thymus (Maillard et al., 2007), while three other groups observed normal frequency while showed a reduced function *in vivo* (Adriani et al., 2007; Humblet-Baron et al., 2007; Marangoni et al., 2007), but all showed a reduced *in vivo* suppression. *Was*^−/−^ nTreg cell failure to control aberrant T cell activation has been also demonstrated *in vivo* in a mouse model of autoimmunity (Humblet-Baron et al., 2007). Moreover, selective advantage of WASP-expressing nTreg cells was shown in a WAS patient with revertant mutation, demonstrating that WASP has a role in nTreg cell fitness (Humblet-Baron et al., 2007). Although the requirement of WASP for nTreg cell functionality has been demonstrated, the role of WASP in these cells is still unclear. Indeed, differently from effector T cells, WASP is not recruited to the IS (Marangoni et al., 2007), thus suggesting a possible role of WASP in TCR signaling of nTreg cells. Moreover, WASP-deficient nTreg cells are also defective in suppressing B cell activation. In fact, it has been shown in *in vitro* studies that nTreg cells from *Was*^−/−^ mice are less efficient in turning off B cell proliferation and this defect is associated with a reduced killing of B cells and significantly decreased secretion of granzyme B by nTreg cells (Adriani et al., 2011). Susceptibility of WAS patients to develop autoimmune diseases can be at least in part explained by nTreg cell dysfunction.

Recent findings have demonstrated that also T effector cells are implicated in tolerance breakdown in WAS. Indeed, in response to restimulation through the TCR, activated T cells can undergo apoptosis, and this event is called restimulation-induced cell death (RICD; Lenardo, 1991; Siegel et al., 2000). RICD process contributes to the maintenance of peripheral immune tolerance by eliminating T cells responding to prolonged presence of antigens, such as self-antigens and persistent pathogen antigens (Critchfield et al., 1994; Ettinger et al., 1995; Weant et al., 2008). In CD4^+^ T cells, RICD is induced by the Tumor Necrosis Factor (TNF) family member Fas ligand (FasL) that is released and binds its receptor Fas in an autocrine fashion (Critchfield et al., 1994; Ettinger et al., 1995; Siegel et al., 2000; Green et al., 2003; Weant et al., 2008). Nikolov and colleagues have shown that WASP is required for T cell apoptosis by RICD. In the absence of WASP, the release of FasL by CD4^+^ T cells is reduced and this is associated to a decreased TCR-mediated apoptosis (Nikolov et al., 2010). Together with nTreg cell defects, these recent findings highlight the role played by effector T cells in the maintenance of T cell tolerance in WAS.

## Regulation of B Cell Tolerance in WAS

In the last years, many studies have assessed the role of B cells in driving autoimmune diseases such as Rheumatoid Arthritis (RA), Multiple Sclerosis (MS), and Systemic Lupus Erythematosus (SLE; Townsend et al., 2010). These data revealed the complex role of B cells that work independently or synergistically with other components of the innate and adaptive immune system to drive autoimmune pathogenesis.

For many years, the functionality of B cells in WAS patients was poorly investigated. The presence of a skewed distribution of serum Ig isotypes (reduced IgM, normal IgG, and elevated IgE and IgA levels) and a reduced or absent antibody production to polysaccharides and other T cell-independent antigens (Golding et al., 1984; Ochs and Thrasher, 2006) represent the first evidences of a defective B cell effector function in WAS patients. This prompted many researchers to investigate more in detail the B cell compartment in WAS, mainly taking advantage of the murine model of the disease (*Was*^−/−^ mice). In the last decade, it has been clearly defined that the lack of WASP causes defects in the cytoskeletal functions of B cells, including adhesion, migration, and homing (Westerberg et al., 2005, 2008; Meyer-Bahlburg et al., 2008). These defects may compromise the capacity of B cells to be properly activated and reach the site of infection contributing to the inability of the immunodeficient host to completely eradicate infectious agents. In this respect, it has been accepted, in particular for PIDs, that chronic immune response due to an incomplete pathogen clearance may favor breakdown of peripheral tolerance. Of note, the complement receptors CD21 (CR2) and CD35 (CR1) are expressed at lower levels on B cells of patients with WAS (Park et al., 2005) contributing to a suboptimal B cell capacity to capture and present opsonized antigens. Additionally, given the critical role of CD21 and CD35 in the negative selection of self-reactive B cells (Prodeus et al., 1998), the altered expression or function of these receptors may affect the maintenance of B cell tolerance in Immune Complex (IC)-mediated autoimmune diseases such as SLE and RA (Erdei et al., 2009).

The fate of self-reactive B cells within the bone marrow and peripheral lymphoid compartment is largely determined by the strength of signal mediated by BCR in response to antigen cross-linking (Nemazee and Burki, 1989; Erikson et al., 1991; Goodnow, 1996). To this regard, reports of a defective BCR activation are controversial. Activation of WASP-deficient B cells was found to be defective after BCR engagement in terms of calcium mobilization in primary B cells isolated from WAS patients and also in WASP-deficient EBV-transformed B cell lines (Simon et al., 1992). However, this defect was not confirmed by Henriquez et al. (1994). More recently, studies performed on *Was*^−/−^ mice showed a normal proliferative response of B cells after stimulation with anti-IgM, LPS, or anti-CD40 (Snapper et al., 1998; Zhang et al., 1999) and a normal or increased class switch (Westerberg et al., 2005). However, the presence of circulating autoantibodies in WAS patients (Dupuis-Girod et al., 2003; Schurman and Candotti, 2003) and in *Was*^−/−^ mice (Humblet-Baron et al., 2007; Nikolov et al., 2010; Becker-Herman et al., 2011; Bosticardo et al., 2011) represents the first evidence of a perturbed B cell tolerance. Very recently, two studies have shown the contribution of B cell intrinsic defects to the pathogenesis of autoimmunity in two different murine models. Indeed, Becker-Herman et al. (2011) observed that female *Was*^+/−^ mice generate anti-nuclear antibodies at rates and titers equivalent to *Was*^−/−^ mice even though heterozygous animals have a normal nTreg cell compartment. Based on this evidence, they demonstrated in mixed BM chimeras, in which only B cells lacked WASP expression, that the selective defect in B cells is sufficient for the generation of autoantibodies. Additionally, they suggested that BCR/Toll-Like Receptor (TLR) co-engagement in *Was*^−/−^ B cells from chimeras could mediate tolerance breakdown, since the loss of Myeloid Differentiation primary response gene 88 (MyD88) signaling abolished the production of anti-dsDNA antibodies, germinal center formation, and development of systemic autoimmune disease (Becker-Herman et al., 2011). More recently, by conditional *WAS* gene deletion in B cells (B/WcKO mice), Recher et al. (2012) observed that WASP deficiency limited to B cells is sufficient to promote autoantibody production and kidney tissue damage in B/WcKO mice.

Overall, these findings highlight the contribution of B cells to the pathogenesis of autoimmunity in WAS and suggest that the B cell autonomous defect could represent a sufficient factor to break tolerance in WAS. However, in addition to the supposed role for TLR signaling in the autoreactivity of *Was*^−/−^ B cells, other mechanisms are potentially involved and need to be further investigated both in mice and humans.

## Future Directions

**Figure 4:**
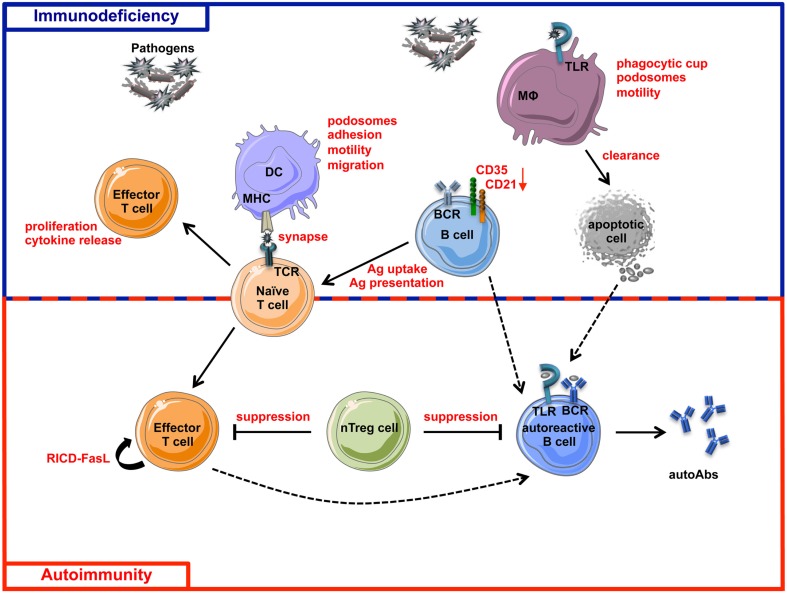
**Schematic view of immunodeficiency and autoimmunity in WAS**. The impairment of both innate and adaptive immune systems is responsible of immunodeficiency (blue box) and autoimmunity (red box) in WAS. Immune cell defects described in WASP-deficient cells are shown in red. The incomplete pathogen clearance is sustained by cytoskeleton and functional defects of macrophages, DCs, T cells, B cells, and their defective interactions. The reduced expression of CD21 and CD35, two complement receptors involved in antigen uptake and presentation and also in negative selection of self-reactive B cells, places B cells at the interface between immunodeficiency and autoimmunity. Defective suppression of WASP-deficient nTreg cells toward both T and B cells contributes to the tolerance breakdown in WAS. Defect in RICD process, resulting in defective effector T cell apoptotic death after TCR restimulation, concurs in the persistence of T cell response to pathogens or self-antigens. Additionally, intrinsic B cell defects contribute to autoimmunity in WAS, probably via a TLR-mediated mechanism. Dashed lines represent hypothetical mechanisms involved in WAS-related autoimmunity. MHC, Major Histocompatibility Complex; RICD, Restimulation-Induced Cell Death; autoAbs, autoantibodies; Ag, Antigen.

WAS is characterized by a very complex spectrum of cellular defects, many of which can predispose patients to the development of autoimmunity (Figure 4).

As described above, defective control of the strength of immune response by nTreg cells, the presence of autoantibodies and potentially autoreactive B cells have been demonstrated in WAS (Bosticardo et al., 2009). However several mechanisms shown to be involved in the pathogenesis of autoimmune diseases still need to be investigated in WAS. iNKT cells have been shown to prevent autoimmune disease in a mouse model of experimental Autoimmune Encephalomyelitis (EAE; Miyamoto et al., 2001; Singh et al., 2001) and uveitis (Oh et al., 2011). Although the mechanisms have not been fully understood, it has been shown that iNKT activation reduces autoimmune symptoms by limiting the development of Th17 cells in a cell contact- and cytokine-dependent manner (Mars et al., 2009). Moreover, in mice, iNKT cells suppress anti-DNA antibody production and reduce autoreactive B cells (Yang et al., 2011), whereas iNKT reduction leads to increased autoreactive B cell activation (Wermeling et al., 2010). Since iNKT cells are reduced in WAS patients and functionally defective in *Was*^−/−^ mice (Astrakhan et al., 2009; Locci et al., 2009) it can be hypothesized that such impairment may also contribute to autoimmunity.

A mechanism contributing to the tolerance breakdown in PIDs is related to the inability of innate immune cells, in particular DCs, to properly activate adaptive immune response (Arkwright et al., 2002). Since DCs have a role in the induction of nTreg cells (Manicassamy and Pulendran, 2011) and DCs lacking WASP are defective in T cell priming (Bouma et al., 2007; Pulecio et al., 2008), it is possible to hypothesize that a defect in nTreg cell induction by DCs might occur in the absence of WASP. Immunodysregulation can be also sustained by overload of pathogen antigens or apoptotic material due to defective clearance by innate immune cells. Antigen overload in fact results in a prolonged immune response, which promotes expansion of Th17 cell subset, playing a central role in many autoimmune diseases, such as MS, RA, and Crohn’s disease (Langrish et al., 2005; Fouser et al., 2008; Isaksson et al., 2009; Sharma et al., 2009). Furthermore, reduced clearance of apoptotic material has been associated to the accumulation of autoantibodies in SLE (Gaipl et al., 2005; Fransen et al., 2012). WASP-deficient DCs are impaired in antigen uptake and migration to secondary lymphoid tissues (Westerberg et al., 2003; de Noronha et al., 2005) suggesting an inefficient pathogen clearance, process that needs to be investigated in *in vivo* models of infection. Moreover, WASP-deficient macrophages are less efficient in uptaking apoptotic cells both *in vitro* and *in vivo* (Leverrier et al., 2001). All together, these findings suggest that dysregulation of Th17 cell activation might contribute to autoimmunity induction in WAS patients, although no evidence has been provided so far to sustain such hypothesis.

Recent studies have demonstrated the key role played by a specific subset of DCs, namely plasmacytoid DCs (pDCs), in the pathogenesis of systemic autoimmune diseases. In particular, IFN-α produced by pDCs upon recognition of foreign nucleic acids via TLR7 and TLR9 contributes to tolerance breakdown in several autoimmune diseases, such as SLE, SS, and psoriasis (Ronnblom, 2011). In these clinical settings, self-nucleic acid-containing ICs trigger TLR7 or TLR9 leading to an uncontrolled pDCs activation. In PIDs, an increased susceptibility to viral infection, in combination with a defective clearance of pathogens, could be the triggering factor of the over-activation of type I IFN pathway. Moreover, cell death induced by viral infection leads to the release and accumulation of self-antigens in the extracellular matrix. Since PID patients, including WAS patients, are highly susceptible to infections and fail to completely eradicate the pathogens, high levels of ICs and activation of the type I IFN system can be expected. Furthermore, increasing evidences highlight the role played by neutrophils in SLE in the induction of type I IFN production. Mature neutrophils are primed *in* *vivo* by type I IFN and die upon exposure to anti-ribonucleoprotein antibodies, releasing neutrophil extracellular traps (NETs) which in turn activate pDCs to produce high levels of type I IFN (Garcia-Romo et al., 2011). Overall, these studies have demonstrated an important role of neutrophils and pDCs in promoting autoimmune diseases and it can be envisaged that these mechanisms may act in the complexity of WAS autoimmunity.

Triggering of autoreactive B cells by self-nucleic acid-containing ICs can be another possible mechanism underlying the production of autoantibodies in WAS. In fact, it is known that self-nucleic acid-containing ICs can activate B cells through synergistic engagement of BCR and TLR7 or TLR9 (Leadbetter et al., 2002; Lau et al., 2005; Chaturvedi et al., 2008), and the loss of MyD88 signaling in *Was*^−/−^ mice abolish the production of anti-dsDNA antibodies (Becker-Herman et al., 2011). The recent findings of B cell intrinsic defect (Becker-Herman et al., 2011; Recher et al., 2012) open a new scenario in tolerance breakdown in WAS although the underlying mechanisms are still unclear. It is known that B cell tolerance is established through central and peripheral checkpoints during B cell maturation which require proper BCR and TLR signaling together with extrinsic factors (Meffre, 2011). The cytoskeleton controls the distribution of the BCR and shapes its signaling (Batista et al., 2010). In particular, the density of actin network inversely correlates with the rate of BCR diffusion and the restriction of BCR diffusion limits signaling. Since WASP is required for actin polymerization and cytoskeletal organization in B cells (Facchetti et al., 1998; Westerberg et al., 2005), it is reasonable to speculate that the threshold of activation might be altered in WASP-deficient B cells. In the bone marrow, receptor editing is the major mechanism aimed at eliminating self-reactive B cells during differentiation (Monroe and Dorshkind, 2007; von Boehmer and Melchers, 2010) by editing autoreactive receptors through secondary rearrangements in light chain loci (Halverson et al., 2004). Abnormal receptor editing is involved in the loss of central B cell tolerance (Ng et al., 2004). Interestingly, alterations in the regulation of secondary recombination events have been reported in BTK-, Interleukin-1 Receptor-associated Kinase 4 (IRAK4)-, and MyD88-deficient patients and in a group of Common Variable Immunodeficiency (CVID) patients with expanded autoreactive CD21^−/low^ B cells (Ng et al., 2004; Isnardi et al., 2008; Meffre, 2011). Given the interaction of WASP with BTK (Cory et al., 1996; Sharma et al., 2009), the involvement of MyD88 signaling in B cell tolerance (Becker-Herman et al., 2011) and the increased frequency of CD21^−^ B cells in WAS patients (Park et al., 2005), it would be worth to investigate whether receptor editing is defective also in the absence of WASP. Furthermore, in the periphery, survival of autoreactive B cells is supported by high levels of BAFF and APRIL, members of the TNF superfamily, found to be increased in several autoimmune diseases (Townsend et al., 2010) and lymphopenic conditions (Cassani et al., 2010). This represents an important mechanism involved in the regulation of peripheral human B cell tolerance that would be interesting to investigate in WAS. Finally, a new function as regulator of immune response has been described for B cells and is mainly mediated by the secretion of IL-10 (Matsushita et al., 2008; Yanaba et al., 2009; Watanabe et al., 2010). Although the origin of regulatory B cells is unclear, MZ B cells (Lenert et al., 2005; Evans et al., 2007) seem to have regulatory functions. Thus, considering the reduction of MZ B cells in *Was*^−/−^ mice (Westerberg et al., 2008; Bosticardo et al., 2011), it would be interesting to investigate whether a defect in regulatory B cell function is a factor contributing to autoimmunity.

In conclusion, together with the defects already described in the literature, these future lines of enquiry underline the greater than expected extent to which the WASP deficiency affects the immune system. Further research is necessary to define the underlying molecular and cellular mechanisms leading to autoimmunity, which represents the main collateral damage caused by WASP deficiency.

## Conflict of Interest Statement

The authors declare that the research was conducted in the absence of any commercial or financial relationships that could be construed as a potential conflict of interest.
